# A crossover randomized comparative study of zofenopril and ramipril on cough reflex and airway inflammation in healthy volunteers

**DOI:** 10.1186/s12997-014-0007-5

**Published:** 2014-12-24

**Authors:** Federico Lavorini, Elisa Chellini, Margherita Innocenti, Giacomo Campi, Colin Gerard Egan, Selene Mogavero, Giovanni A Fontana

**Affiliations:** Department of Experimental and Clinical Medicine, University of Florence, Largo Brambilla 3, 50134 Firenze, Italy; Primula Multimedia S.r.L., Via Giuseppe Ravizza 22/B, 56121 Pisa, Italy

**Keywords:** Zofenopril, Ramipril, Cough, ACE-inhibitors, Airway inflammation

## Abstract

**Background:**

Persistent dry cough is a well known unwanted effect of Angiotensin-Converting Enzyme inhibitors (ACE-i). Animal studies have shown that the ACE-i zofenopril has a less tussigenic effect compared to the widely used ACE-i ramipril. The aim of this study was to compare cough sensitivity to inhaled tussigens, as well as spontaneous cough in response to the administration of zofenopril and ramipril in healthy volunteers; pharmacokinetic (PK) data of both zofenopril and ramipril, as well as their respective active forms, zofenoprilat and ramiprilat, was also collected.

**Methods:**

Forty healthy volunteers were enrolled in a randomized crossover study. Patients were administered zofenopril calcium salt (test drug) coated tablets, 30 mg daily dose or ramipril (reference drug) tablets, 10 mg daily dose, for 7 consecutive days in two periods separated by a 21-day wash-out period. Cough sensitivity to capsaicin and citric acid was assessed as the concentration of each tussigenic agent causing at least 2 (C2) or 5 coughs (C5); spontaneous cough was also monitored throughout the study. PK parameters of zofenopril, ramipril and their active forms, were collected for each of the two study periods. Airway inflammation, as assessed by fractional exhaled nitric oxide (FeNO) and bradykinin (BK) levels, were measured prior to and following each treatment period.

**Results:**

Ramipril, but not zofenopril, increased (p < 0.01) cough sensitivity to both tussigenic agents as assessed by C2. With citric acid, C5 values calculated after both ramipril and zofenopril administration were significantly (p < 0.05 and p < 0.01, respectively) lower than corresponding control values. With both ACE-i drugs, spontaneous cough was infrequently reported by subjects.

Zofenopril/zofenoprilat PK analysis showed higher area under the curve of plasma concentration, τ values (ng/ml x h) than ramipril/ramiprilat (zofenopril vs. ramipril, 84.25 ± 34.47 vs. 47.40 ± 21.30; and zofenoprilat vs. ramiprilat, 653.67 ± 174.91 vs. 182.26 ± 61.28).

Both ACE-i drugs did not affect BK plasma levels; in contrast, ramipril, but not zofenopril, significantly increased control FeNO values (from 24 ± 9.6 parts per billion [PPB] to 33 ± 16 PPB; p < 0.01).

**Conclusions:**

Zofenopril has a more favourable profile when compared to ramipril as shown by a reduced pro-inflammatory activity and less impact on the cough reflex.

## Introduction

Angiotensin-Converting Enzyme inhibitors (ACE-i) were originally developed to target hypertension but now have additional clinical indications such as congestive heart failure, left ventricular dysfunction, atherosclerotic vascular disease and diabetic nephropathy [1]. It is purported that they alter the balance between the vasoconstrictive, salt-retentive, and hypertrophic properties of angiotensin II (Ang II) and the vasodilatory and natriuretic properties of bradykinin (BK) and alter the metabolism of a number of other vasoactive substances [1].

Zofenopril is indicated for the treatment of mild to moderate essential hypertension and of patients with acute myocardial infarction [2]. After oral administration, zofenopril is completely absorbed and converted into its active metabolite, zofenoprilat, which reaches peak blood levels after 1.5 h [3]. The plasma ACE activity is suppressed by 74.4% at 24 h after administration of single oral doses of 30 mg zofenopril calcium, the usual effective daily dose.

Ramipril is indicated for the treatment of hypertension, symptomatic heart failure, mild renal disease, for cardiovascular prevention and secondary prevention after acute myocardial infarction. Based on urinary recovery, the extent of absorption is at least 56%. Peak plasma concentrations of ramiprilat, the sole active metabolite of ramipril, are reached 2-4 h after intake. The peak antihypertensive effect of a single dose is usually reached 3-6 h after oral administration and usually lasts for 24 h [4].

Dry, persistent cough is a well-recognized side effect of ACE-i, the mechanism of which is not completely understood [5]. The incidence of ACE-i induced cough is variable, and ranges between 3-35% among various studies [5,6]. Interestingly, some lines of evidence seem to suggest that coughing induced by the ACE-i zofenopril has a lower prevalence compared to other ACE-i [5]. The inflammatory mediators BK and substance-P are known to be involved, since they accumulate in the upper respiratory tract or lung after the enzyme is inhibited and fails to degrade them [6]. BK also stimulates the production of prostaglandins which, when accumulating, also seem to induce cough [6].

A study performed on guinea pigs showed that zofenopril administration did not increase citric-acid induced cough, as opposed to ramipril, which augmented it by 40-60% [7]. Similar results were obtained in rabbits, where ramipril, but not zofenopril, increased the cough response induced by both mechanical and chemical airway stimulation [8].

The aim of this study was to assess changes in the sensitivity of the cough reflex, both spontaneous and induced by tussigens, in healthy volunteers administered with zofenopril and ramipril. This analysis was coupled with the analysis of the pharmacokinetics (PK) of the two administered drugs, the collection of airway inflammation data by means of a simple, non invasive method such as the measurement of the fractional exhaled nitric oxide (FeNO) and the assessment of serum BK.

## Methods

### Study subjects

The present study included male (n = 17) and female (n = 23) healthy volunteers aged between 18 and 55 years (Table 1). Pregnant or breast-feeding women, subjects abusing alcohol or drugs, those using any prescription or over-the-counter medication on a regular basis, history of gastrointestinal, renal, hepatic, pulmonary or cardiovascular disease, epilepsy, asthma, diabetes, psychosis or glaucoma, smokers of more than 10 cigarettes/day, subjects with known allergy to ACE-i, and subjects following abnormal diets or practicing vegetarians, since these conditions may influence drug PK [9], were not eligible for inclusion in the study. Self-reported medical conditions were compared/cross referenced with previous and current medical records. In addition, to minimize potential confounder effects in FeNO measurement, subjects could not consume fresh grapefruit or drink caffeine-containing beverages from 24 h prior to and until last blood sampling time after each administration, abstain from smoking 24 h beforehand, avoid alcoholic beverages and strenuous physical exercise. The study protocol adhered to the recommendations of the Declaration of Helsinki for Human Experimentation and was approved by the local ethics committee; informed consent was obtained from each participant.

**Table 1:** **Demographic and clinical characteristics of the 40 healthy volunteers (23 females) who participated to the study**

**General**	
Age (years)	37.4 ± 9.8
Height (cm)	170.5 ± 10.8
Weight (Kg)	71.2 ± 14.5
BMI (Kg/m^2^)	24.2 ± 3.2
**Vital signs**	
Systolic BP (mmHg)	121 ± 9.5
Diastolic BP (mmHg)	78.1 ± 6.0
Heart rate (beats/min)	62.8 ± 8.4
Body temperature (°C)	36.4 ± 0.3
Respiratory rate (breaths/min)	10.7 ± 0.6

Data presented as mean ± SD or number and percentage in parentheses. **BMI**, body mass index; **BP**, blood pressure.

### Study design and treatments

This was a repeated-dose, balanced, two-sequence, two-period, two-treatment, non-placebo controlled, randomized, crossover design study. Since this study examined two ACE-i agents with similar characteristics and was performed using an open design, blinding was not necessary. Subjects were randomly assigned to receive zofenopril calcium salt (test drug) coated tablets, 30 mg daily dose, or ramipril (reference drug) tablets, 10 mg daily dose, for 7 consecutive days followed by a 21 (±2) day wash-out period, after which another 7-day period would follow where subjects would receive the other treatment (Table 2). The administered doses were those used for treatment of hypertension and which would yield a similar percentage of responders [2,4].

**Table 2:** **Study assessments and timetable**

	**1st treatment period**							**Wash-out**	**2nd treatment period**						
Day(s)	1	2	3	4	5	6	7	8-29(±2)	30	31	32	33	34	35	36
Drug dosing	x	x	x	x	x	x	x		x	x	x	x	x	x	x
Vital signs recordings	x						x		x						x
Capsaicin and citric acid challenges	x						x		x						x
Spontaneous cough recordings at home	From day 1 to 7								From day 30 to 36						
FeNO measurement^a^	x						x		x						x
Assessment of pre-dose PK parameters	x		x		x	x	x		x		x		x	x	x
Assessment of post-dose PK parameters^b^							x								x
Pre-dose BK measurements	x						x		x						x
Post-dose BK measurements^c^							x								x
AE monitoring	From day 1 to 7									From day 30 to 36					

**FeNO**, fractional exhaled nitric oxide; **AE**, adverse event; **PK**, pharmacokinetic; **BK**, bradykinin; ^a^ FeNO assessments were performed at pre-dose, 1.5 h and 5.5 h post-dose; ^b^Blood samples obtained 20', 40', 1 h, 1 h30', 2 h, 3 h, 4 h, 5 h, 6 h, 8 h, 10 h, 12 h, 16 h, 24 h after drug administration; ^c^ measurement performed 40', 1 h, 2 h, 4 h, 6 h, 10 h, 16 h, and 24 h after drug administration.

### Assessment of cough sensitivity

Capsaicin and citric acid cough challenges were performed prior to and following each 7-day treatment period (Table 2). Cough sensitivity was assessed as the lowest capsaicin or citric acid concentrations causing at least 2 (C2) or 5 coughs (C5), provided that cough was still present following inhalation of the next tussigenic concentration [10]. C2 and C5 values were converted to logC2 and logC5, respectively, for analysis. Concentrations of both capsaicin and citric acid were prepared according to standard procedures [10], nebulized by a jet nebulizer (DeVilbiss 646, DeVilbiss Health Care Inc., Somerset, PA) driven by compressed air (8 L/min), and inhaled for 1 min during normal tidal breathing. Volunteers undergoing cough challenges were specifically instructed not to attempt to suppress coughs and not to talk immediately after inhalation of the tussigenic agent. In addition, subjects were given the following instruction: “allow yourself to cough if you need to, and as much as you need to”. Subjects were also requested to note on a diary the occurrence of spontaneous cough during the two 7-day treatment periods, using a verbal scale.

### Pharmacokinetic and bradykinin analysis

Blood samples for the measurement of PK parameters and for BK determination were obtained at pre-dose and after drug administration for each of the two study periods (Table 2). For both zofenopril and ramipril, and their respective active forms, zofenoprilat and ramiprilat, the lowest (C_min_) plasma concentration in the “τ” period (i.e. the 24 h interval after drug administration on day 7), and the area under the curve of plasma concentration (AUC_ss,τ_ ) in the period “τ”, were determined. Repeated pre-dose PK variables determination was performed in order to establish baseline variability.

### Assessment of airway inflammation

Serial measurements of FeNO were performed at baseline and following (1.5 h and 5.5 h ± 30 min) each 7-day treatment period with ramipril or zofenopril (Table 2). FeNO measurements were always performed before cough challenges using a standardized single-breath method with an electrochemical analyzer (HypAir FeNO system, Medisoft, Sorinnes, BE). Subjects were seated (with no nose clip), and exhaled to residual volume, inserted the mouthpiece, inhaled to total lung capacity, then exhaled for 10 seconds at a constant flow rate of 0.05 L/s ± 10%. The end-point of measurement was considered when a plateau of at least 4 seconds was observed. Exhalations were repeated after a 30-second period of relaxation until 3 independent FeNO values with ≤10% variation were obtained [11].

### Statistical analysis

Based on the results of previous investigations [12,13], the sample size of 40 patients was chosen to design the study to have a 90% statistical power of detecting a mean change in capsaicin LogC5 of 1.64 μM with a LogC5 standard deviation of 1.91 μM.

Cough sensitivity to both citric acid and capsaicin observed after administration of the test and the reference drug were compared by means of non-parametric analysis of variance for repeated measures. Spontaneous cough occurring during the two treatment periods was only qualitatively assessed, as the prevalence was expected to be low or very low. AUC_ss,τ_ PK parameters were calculated from the individual concentration-time data by using the program WinNonlin software (Pharsight Corporation, Mountain View, CA, USA) and summarized by treatment by means of descriptive statistics, in order to determine mean and standard deviation values. Paired t-test was used to compare mean FeNO values recorded after administration of the test and the reference drug. Statistical analyses were performed by using GraphPad Prism, version 3.02 (GraphPad Software, Inc. La Jolla, CA); sample size and power calculations were performed by using a dedicated software (nQuery Advisor, release 2.0, Los Angeles, CA). A p value <0.05 was considered statistically significant.

## Results

All subjects completed the study. Adverse events of mild intensity were reported by 13 subjects (5 after ramipril and 8 after zofenopril) and included headache, vomit, backache and vertigo. Vital signs (blood pressure, heart rate, body temperature, respiratory rate) were not significantly affected by the two treatments.

### Cough sensitivity

**Figure 1:**
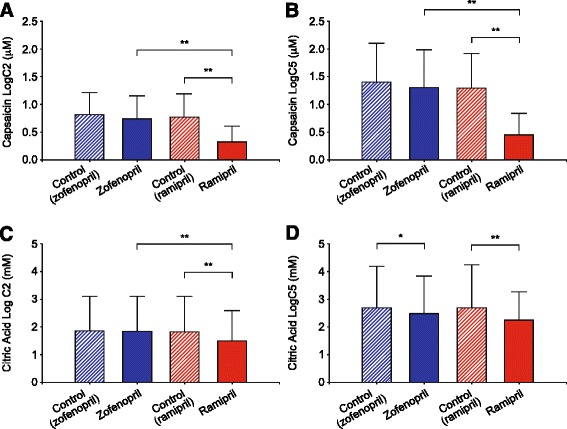
**Mean (±SD) Log values of the capsaicin (A, B) and the citric acid (C, D) concentration causing at least two (C2) and five (C5) coughs recorded in control conditions (pre-treatment, cross hatched bars) and after a 7-day treatment (filled bars) with zofenopril (blue bars) or ramipril (red bars) in 40 normal volunteers.** *, p < 0.05; **, p < 0.01.

With capsaicin, mean (±SD) control LogC2 values observed prior to zofenopril (0.81 ± 0.42 μM) and ramipril (0.78 ± 0.41 μM) administration did not significantly differ (Figure 1A). However, ramipril administration consistently lead to an increase in cough sensitivity following inhaled capsaicin, as shown by the significant reduction in LogC2 (0.33 ± 0.28 μM, p < 0.01) compared to control values. In contrast, zofenopril administration resulted in only a slight and non-significant decrease in capsaicin LogC2 (0.75 ± 0.40 μM).Overlapping results were observed with capsaicin LogC5 values (Figure 1B). Prior to drug administration, LogC5 values for zofenopril and ramipril controls were similar (1.4 ± 0.72 μM and 1.3 ± 0.63 μM, respectively); they were reduced to 1.3 ± 0.68 (non significant, [ns]) after zofenopril and to 0.45 ± 0.38 μM (p < 0.01) after ramipril treatment.

With citric acid, mean (±SD) control LogC2 values prior to zofenopril and ramipril administration (1.85 ± 1.24 mM and 1.80 ± 1.28 mM, respectively) did not significantly differ (Figure 1C). On the other hand, ramipril administration significantly increased cough sensitivity to inhaled citric acid, as shown by the significant reduction in LogC2 (1.48 ± 1.09 mM, p < 0.01) compared to control values. In contrast, zofenopril administration lead to only slight and inconsistent changes in citric acid LogC2 values (1.81 ± 1.27 mM, ns). Control LogC5 values of zofenopril and ramipril did not significantly differ (Figure 1D). However, both zofenopril and ramipril significantly decreased LogC5 values to citric acid, from 2.69 ± 1.88 mM to 2.51 ± 1.57 mM with zofenopril (p < 0.05) and from 2.67 ± 2.01 mM to 2.23 ± 1.04 mM with ramipril (p < 0.01). The reduction in citric acid LogC5 induced by zofenopril did not significantly differ from that provoked by ramipril.

During treatment with zofenopril, 7 volunteers out of 40 recorded at least 1 spontaneous coughing episode, with a total of 36 distinct coughing episodes. With ramipril, 9 volunteers recorded at least 1 coughing episode, with a total of 24 distinct coughing episodes.

### Pharmacokinetics

**Figure 2:**
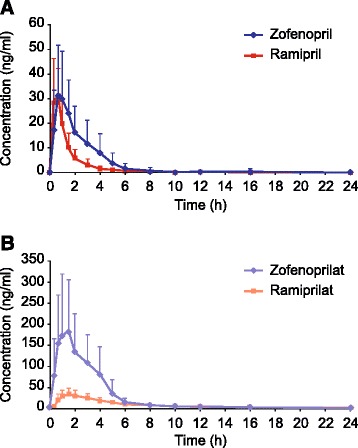
**Pooled plasma-concentration/time profiles of zofenopril/ramipril (A) and zofenoprilat/ramiprilat (B) obtained in 40 volunteers.** Data presented as mean ± SD.

At baseline, plasma zofenopril or ramipril and their respective active forms (zofenoprilat/ramiprilat) were not detected (Figure 2); the time course of plasma concentration after administration of either zofenopril or ramipril was qualitatively similar for both drugs and their respective active forms (Figure 2). Mean (±SD) AUC_ss,τ_ values (ng/ml x h) were 84.25 ± 34.47 for zofenopril, 653.67 ± 174.91 for zofenoprilat, 47.40 ± 21.30 for ramipril, and 182.26 ± 61.28 for ramiprilat. Both test and reference drugs C_min_ was 0, whereas traces of the active compounds were found, with C_min_ values for zofenoprilat and ramiprilat being 1 ± 1.29 and 1.25 ± 0.39 respectively.

### Airway inflammation

**Figure 3:**
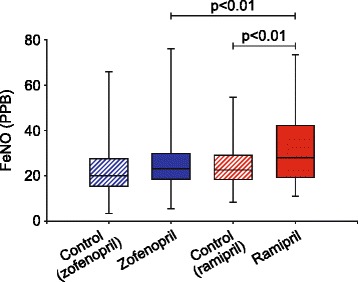
**Box and whiskers plots illustrating changes in fractional exhaled nitric oxide (FeNO) recorded in control conditions (pre-treatment) and after a 7-day treatment period with zofenopril or ramipril in 40 normal volunteers.** Data presented as median, 25th/75th percentiles and maximum/minimum recorded values. **PPB**, parts per billion.

Mean (±SD) FeNO control values (expressed in parts per billion, PPB) obtained prior to zofenopril (22 ± 12 PPB) and ramipril (24 ± 9.6 PPB) administration did not significantly differ (Figure 3). Administration of zofenopril lead to a slight and non-significant increase in mean FeNO (26 ± 12 PPB), whereas administration of ramipril resulted in marked increases in FeNO (33 ± 16 PPB) compared to both the corresponding control condition and the mean FeNO values recorded following zofenopril administration (p < 0.01 for both treatments, Figure 3).

### Bradykinin analysis

**Figure 4:**
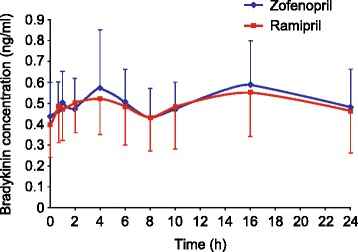
**Pooled bradykinin plasma concentration/time profiles of all volunteers obtained after administration of either zofenopril, 30 mg (blue line) or ramipril, 10 mg (red line).** Data presented as mean ± SD.

Figure 4 shows the pooled BK plasma concentration/time profiles of the 40 volunteers, obtained on day 7 of either treatment period. No difference was found for BK levels after administration of zofenopril or ramipril. Pre-dose levels of BK on day 1 of either treatment period were 0.44 ± 0.17 ng/ml and 0.42 ± 0.16 ng/ml, respectively for zofenopril and ramipril, not different from pre-dose levels on day 7.

## Discussion

The main findings from this study suggest that short-term administration of therapeutic doses of zofenopril and ramipril have a different impact on the functionality of the cough reflex, with ramipril markedly affecting the cough sensitivity – as assessed in terms of C2 and C5 - to both capsaicin and citric acid, whereas zofenopril provoked only a minimal, albeit significant, decrease in citric acid C5. These results reinforce and extend similar observations previously obtained in animal models [7,8] and in healthy volunteers [14]. Although coughing is a well recognized, unwanted effect of ACE-i drugs [6], the mechanism by which these agents cause cough remains unclear. The effect may be related to a cascade of effects beginning with the accumulation of kinins, followed by arachidonic acid metabolism and the production of nitric oxide [15]. ACE inhibition can block BK dehydrogenase, the enzyme responsible for BK breakdown, and may lead to the accumulation of BK in the airways. BK has many local effects, including the release of histamine from mast cells, and also interferes with locally produced neurotransmitters, such as substance-P and neuropeptide-Y which are released by vagal C-fibres and are known to have irritant effects on the bronchial mucosa and increase cough responses [8]. Another factor that has been reported to be involved in cough induction is prostaglandin synthesis in the airways, since prostaglandins act locally as inflammatory agents [16]. Prostaglandin E2 stimulates airway sensory fibres possibly involved in cough mediation (as does BK), resulting in cough [17]. On the other hand, treatment with a prostaglandin synthetase inhibitor may alleviate cough in affected patients [18].

Other factors that may explain the observed differences between zofenopril and ramipril in inducing cough reflex may be attributed to differences in the pharmacokinetic profiles and differences in the ability of tissue and blood esterases to hydrolyse their active metabolites, zofenoprilat and ramiprilat respectively [19,20]. In this regards, a previous study has shown that the ramiprilat-ACE complex is very stable and dissociates more slowly compared with complexes formed by the enzyme and other ACE inhibitors [21].

Spontaneous cough after either ACE-i drugs was infrequently reported by subjects, likely because it may take weeks or even months to develop ACE-i-associated cough [5].

In the present study, BK levels did not differ after administration of zofenopril or ramipril; thus the less tussigenic property of zofenopril compared to ramipril cannot be explained by the elevated BK levels following ACE-i administration. However, as shown in a previous in-vivo study [22], the capability of zofenopril to stimulate the production of prostaglandins, either directly or by inhibiting BK metabolism, is less than that of other ACE-i.

It has also been previously shown that in normotensive volunteers enalapril is capable of increasing FeNO within a few hours [23]. Furthermore, it is unclear whether ‘ACEi-induced cough’ as a clinical problem is directly related to changes in FeNO, as the effects were not directly evaluated in hypertensive patients, but only in healthy volunteers. Evidence suggests that hypertensive patients have reduced baseline FeNO levels [23,24] and did not show FeNO increase in response to enalapril administration, unlike normotensive subjects [23]. Additional studies in hypertensive subjects are still needed to clarify this.

It is likely that the activation of sensory airway terminal by ACE-i agents may result in an enhancement of the cough reflex and, eventually, in a decrease of the stimulus intensity required to evoke cough, thus explaining the present findings of an increased cough sensitivity in normal subjects under treatment with therapeutic doses of ramipril. The fact that zofenopril affected cough sensitivity to a much lesser extent compared to ramipril is in keeping with the notion of a less pronounced stimulatory effect on prostaglandin production and/or inhibitory activity on BK breakdown by zofenopril [7]. Further studies on the co-administration of an ACE-i and a COX inhibitor could help clarify the tussigenic role of prostaglandins with and without ACE-i.

To our knowledge, this is the first study to evaluate airway inflammation, as detected by a non invasive method such as the assessment of FeNO, in normal subjects undergoing short-term treatment with ACE-i. Results show that ramipril, but not zofenopril, causes airway inflammation. The same mechanisms as for cough induction may also be invoked to account for a lack of any significant change in FeNO observed following zofenopril, but not ramipril administration in our subjects. Again, this finding points to the possibility that these agents must have a different impact on arachidonic acid metabolism and BK breakdown.

In the present study we examined AUC_ss,τ_ values and these were quantitatively higher with zofenopril/zofenoprilat compared to ramipril/ramiprilat. These data suggest that a longer lasting activity is to be expected with zofenopril.

This study performed in normal subjects was planned and carried out following the crossover two-treatment, two-sequence, two-product design. This meant that all subjects experienced both treatments, and the crossover guaranteed a good degree of comparison of the two ACE-i, namely zofenopril, test drug, and ramipril, reference drug in this study.

A limitation of the present study is the absence of a placebo arm, and the question arises as to whether the observed differences in cough sensitivity and airway inflammation after ACE-i treatments are a true treatment effect. A placebo effect has been observed in several cough clinical trials, and up to 85% of the efficacy of some cough medicines can be attributed to a placebo effect [25]. However, the presence of significant plasma concentration levels of both ACE-i drugs points at the possibility that the results obtained in the present study are related to treatment, rather than to a placebo effect.

In conclusion, findings of the present study suggest that zofenopril possesses a more favourable therapeutic profile when compared to ramipril, mainly consisting of a lower impact on the sensitivity of the cough reflex, as detected by widely used laboratory methods, and lack of a significant pro-inflammatory action at the level of the airways. The more tolerable profile of zofenopril is coupled with an equivalent or even better efficacy than ramipril in the prevention and treatment of cardiovascular diseases, as evidenced by several head-to-head trials [26-28].
